# The Molecular Link Between TDP-43, Endogenous Retroviruses and Inflammatory Neurodegeneration in Amyotrophic Lateral Sclerosis: a Potential Target for Triumeq, an Antiretroviral Therapy

**DOI:** 10.1007/s12035-023-03472-y

**Published:** 2023-07-14

**Authors:** Megan Dubowsky, Frances Theunissen, Jillian M. Carr, Mary-Louise Rogers

**Affiliations:** 1 College of Medicine and Public Health and Flinders Health and Medical Research Institute, Flinders University, Bedford Park, SA Australia; 2 Perron Institute for Neurological and Translational Science, Nedlands, WA Australia; 3 Centre for Molecular Medicine and Innovative Therapeutics, Murdoch University, Murdoch, WA Australia

**Keywords:** Amyotrophic lateral sclerosis, Motor neuron disease, TDP-43, Endogenous retrovirus, HERV-K neuroinflammation, Antiretroviral therapy, Triumeq

## Abstract

Amyotrophic lateral sclerosis (ALS), also known as motor neuron disease (MND), is a progressive neurological disorder, characterised by the death of upper and lower motor neurons. The aetiology of ALS remains unknown, and treatment options are limited. Endogenous retroviruses (ERVs), specifically human endogenous retrovirus type K (HERV-K), have been proposed to be involved in the propagation of neurodegeneration in ALS. ERVs are genomic remnants of ancient viral infection events, with most being inactive and not retaining the capacity to encode a fully infectious virus. However, some ERVs retain the ability to be activated and transcribed, and ERV transcripts have been found to be elevated within the brain tissue of MND patients. A hallmark of ALS pathology is altered localisation of the transactive response (TAR) DNA binding protein 43 kDa (TDP-43), which is normally found within the nucleus of neuronal and glial cells and is involved in RNA regulation. In ALS, TDP-43 aggregates within the cytoplasm and facilitates neurodegeneration. The involvement of ERVs in ALS pathology is thought to occur through TDP-43 and neuroinflammatory mediators. In this review, the proposed involvement of TDP-43, HERV-K and immune regulators on the onset and progression of ALS will be discussed. Furthermore, the evidence supporting a therapy based on targeting ERVs in ALS will be reviewed.

**Graphical Abstract:**

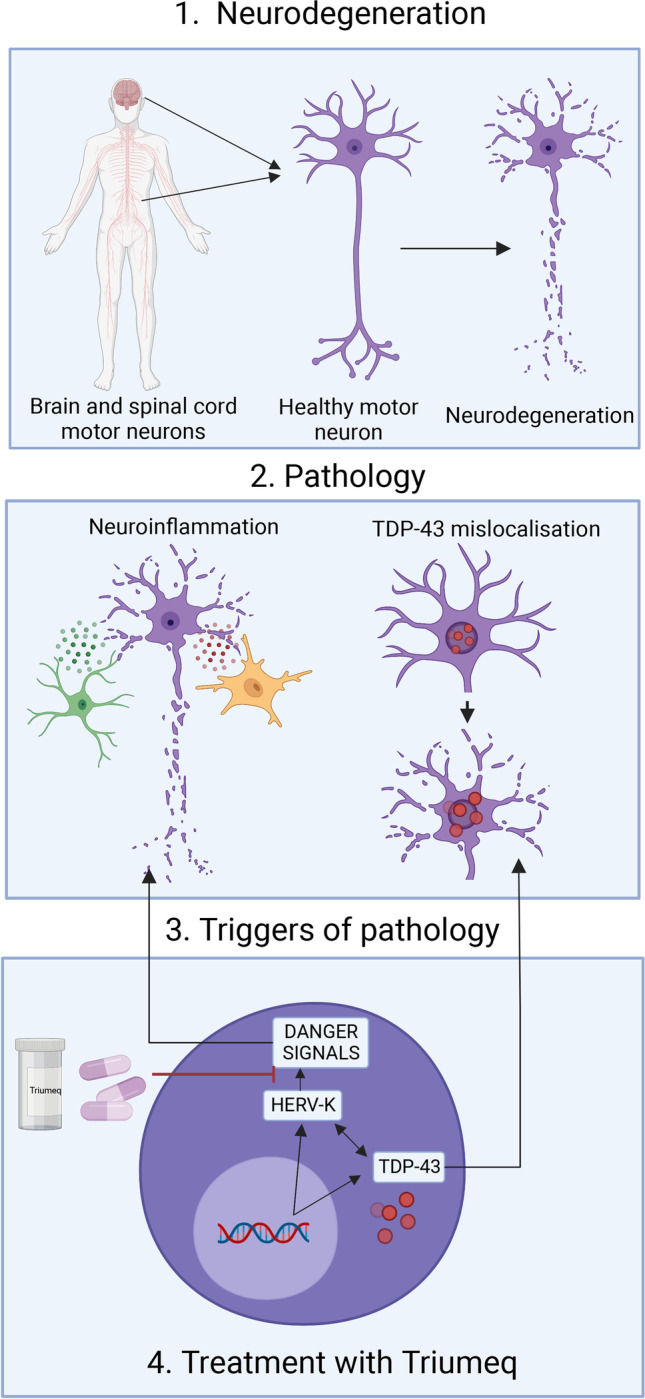

## Introduction

Amyotrophic lateral sclerosis (ALS), also known as motor neuron disease (MND), is a progressive and fatal neurological disorder, characterised by the death of both upper and lower motor neurons in the primary motor cortex and spinal cord [1, 2]. Death of motor neurons results in muscular atrophy leading to the eventual inability to activate muscles of the body and control vital functions, such as respiration [2, 3]. ALS is considered a highly complex and heterogenous disease where both the clinical features and disease progression can vary between patients [4]. This can make diagnosing ALS extremely challenging, with patients often waiting a year from symptom onset to diagnosis. At present, the underlying cause of ALS remains elusive, and with no cure or disease-modifying therapy, the typical life expectancy lies between 2 and 5 years from diagnosis [5–7].

A hallmark of ALS pathology is cytoplasmic TDP-43 aggregation. While only 4% of familial ALS cases can be attributed to mutations in *TARDBP*, 95% of ALS cases have a cellular TDP-43 pathology. Cytoplasmic TDP-43 aggregation is present in both sporadic and familial ALS with the exception of those with familial ALS caused by SOD1 mutations [8]. In healthy neurons and non-neuronal cells including astrocytes and microglia, TDP-43 is localised with the nucleus [9]. However, TDP-43 inclusions are present within the cytoplasm of neurons and some glial cells in ALS patients at autopsy [10, 11]. Cytoplasmic TDP-43 inclusions have been implicated as a major player in the initiation and progression of neurodegeneration [12, 13].

Both central and peripheral inflammation have been well established in ALS, with this pathology encompassing increased levels of inflammatory cytokines including tumour necrosis factor α (TNF-α) and interleukins (IL-1β, IL-4, IL-6 and IL-10) the involvement of non-neuronal cells including activation of microglia and astrocyte dysfunction, and T lymphocyte infiltration into the central nervous system (CNS) [14–17].

Expression of endogenous retroviruses (ERVs) has also been implicated in neurodegenerative mechanisms in ALS [18]. ERVs are remnants of ancient viral infection that became fixed within the genome. Due to mutations and deletions, ERVs were thought to be inactive and lack coding capacity [19, 20]. However, their involvement in ALS has been investigated with ERV transcripts discovered in the brain tissue from ALS patients [21] and with elevated reverse transcriptase (RT) levels in the serum and cerebral spinal fluid (CSF) of ALS patients [22]. ERV reactivation and TDP-43 proteinopathy have both been associated with increased inflammation, and hence a link between ERVs, TDP-43 and inflammation is thought to be a driving factor behind neurodegeneration in ALS [23, 24]. The mechanisms of TDP-43, neuroinflammation and ERVs in ALS will be the focus of this review, and the interplay between these three mechanisms will be discussed. The rationale for the use of antiretroviral therapy for ALS based on the involvement of ERVs will also be reviewed.

## Genetics and Pathophysiology of ALS

Inherited genetic causes, known as familial ALS, account for 10% of cases, while the remaining 90% are regarded as sporadic [25]. Interestingly, twin studies have revealed that there is 65% heritability in familial ALS and up to 37% heritability for relatives of an individual with no known genetic risk, demonstrating a strong genetic influence for developing ALS [26]. Within familial ALS, 60% of familial cases are accounted for by 4 genes including GGGGCC hexanucleotide repeats in chromosome 9 open reading frame 72 (*C9orf72*; 40%) [27, 28], missense mutations in superoxide dismutase 1 (*SOD1*; 12%) [29], point mutations in TAR DNA binding protein 43 (*TDP-43*; 4%) [10] and point mutations in the fused in sarcoma gene (*FUS*; 4%) [30, 31]. The remaining 40% of familial cases are thought to be explained by rare gene mutations in a number of ALS linked genes including TANK-binding kinase 1 (*TBK1*), NIMA-related kinase 1 (*NEK1*) and additional yet-to-be identified genes [15, 32].

The identification of genes involved in ALS pathology has helped elucidate some of the mechanisms underlying neurodegeneration including protein accumulation from *SOD1* and *TARDBP* [15, 33, 34]. Other known neurodegenerative mechanisms include excitotoxic mechanisms as a result of increased glutamate levels [35], impaired protein homeostasis resulting in misfolded protein accumulation [36], axonal transport dysfunction [37], mitochondrial dysfunction [38], neuroinflammation [39] and TDP-43 pathology [13].

## TDP-43 Mislocalisation is Associated with Progression of ALS

### TDP-43 Structure and Function

TDP-43 contains a nuclear localisation sequence, a nuclear export signal, two highly conserved RNA recognition motifs (RRM1 and RRM2) and a glycine-rich C-terminal domain [33]. The nuclear localisation sequence and nuclear export signal enable the transport of TDP-43 between the nucleus and the cytoplasm through importin-α [40]. The RNA recognition motifs enable the identification and binding of the TDP-43 to RNA, while the glycine-rich domain of TDP-43 is critical for protein–protein interactions [11, 41, 42]. The functions of TDP-43 occur predominately within the nucleus where TDP-43 binds to DNA and RNA and is involved in transcriptional regulation, RNA splicing and stability and transport of mRNA [43, 44]. TDP-43 also has cytoplasmic functions including translation, mRNA transport and stress granule formation [12, 45, 46]. TDP-43 was first described as a transcription factor that regulates the transcription of the human immunodeficiency virus (HIV) trans-activation response (TAR) element to repress HIV-1 transcription [47]. Since then, TDP-43 has been identified as a transcriptional repressor involved in the repression of a spermatid-specific gene *ACRV* with the promotor region containing TDP-43 binding sites [48]*.* Lalmansingh et al. [49] experimentally identified the role of TDP-43 in transcriptional repression of *ACRV*, localising the repressor activity to the RRM1 region of TDP-43. Mutations in TDP-43 causing dysfunctional RRM1 mitigated the repressor activity of TDP-43. In addition, TDP-43 was also found to be involved in the alternative splicing of human cystic fibrosis transmembrane conductance regulator (*CFTR*) exon 9 [50] and human survival of motor neuron 2 (*SMN2*), a gene associated with sporadic ALS [51]. Furthermore, TDP-43 also regulates the alternative splicing of ciliary neurotrophic factor receptor (CNTFR), a protein that is implicated in neurodegeneration [52–54].

In 2006, the initial link between TDP-43 and familial ALS was identified through the presence of ubiquitinated and hyper-phosphorylated TDP-43 inclusions in histological sections of the cortex and spinal cord, which are now considered a hallmark pathology of ALS [10, 55]. Since this discovery, approximately 35 ALS-causing mutations related to TDP-43 have been discovered [56, 57]. Most of these mutations are missense mutations located within the glycine-rich domain of the protein with only 4 mutations in the RRM1 and RRM2 domains [33, 58]. These single nucleotide mutations interrupt the function of the glycine-rich C-terminal domain, impairing protein–protein interactions including its direct binding to members of the heterogenous nuclear ribonucleoprotein family, which are involved in alternative splicing. Some mutations have been proposed to alter TDP-43 phosphorylation sites, which has been hypothesised to result in the accumulation of protein aggregates and hyperphosphorylation of TDP-43, potentially involved in neurodegeneration [59, 60].

Another potential mechanism contributing to the formation of TDP-43 protein aggregates is via the disruption to the autoregulatory activity of TDP-43. Under normal conditions, TDP-43 self-regulates its own expression through binding to 3′UTR sequences in its own mRNA, promoting degradation to decrease TDP-43 levels [61]. However, this self-regulating negative feedback loop is affected by non-functional TDP-43 aggregates that are unable to bind the mRNA, increasing TDP-43 levels and perpetuating the neurodegenerative process [61].

### TDP-43 Involvement in Neurodegeneration

One hypothesis for TDP-43 linked neurodegeneration is a loss-of-function of nuclear TDP-43. DNA damage and an impaired DNA repair system have been proposed as a cause of neurodegeneration from nuclear loss of TDP-43 [62, 63]. In neuronal SH-SY5Y cells with an inducible TDP-43 depletion system, an increase in unrepaired DNA double-strand breaks correlated with the level of TDP-43 depletion in a dose-dependent manner and was independent of cytoplasmic aggregations [63]. Similarly, changes in expression of genes related to DNA damage have been observed in pathology-affected neurons from neocortex brain tissue from ALS patients and associated with loss of nuclear TDP-43 [64]. Loss of nuclear TDP-43 has been suggested to cause neurodegeneration by altering RNA processing as determined by altered patterns of gene splicing in shRNA-mediated TDP-43 knock-down in NSC34 cells [65].

In addition to loss-of-function TDP-43-associated neurodegeneration, a gain of toxicity from the cytoplasmic TDP-43 inclusions may also induce neurodegeneration. Barmada et al. [66] used rat primary cortical neurons transfected with constructs encoding human ALS-linked mutant TDP-43 or wild-type TDP-43, to identify the effects of TDP-43 nuclear clearance and cytoplasmic aggregation on neuronal death. Transfection of mutant TDP-43 increased the presence of cytoplasmic TDP-43 aggregates compared to the wild-type TDP-43 transfected cells, and the level of cytoplasmic TDP-43 was an accurate predictor of cell death, indicating gain-of-function toxicity [12, 66]. Gain-of-function toxicity from cytoplasmic TDP-43 occurs through both the disruption of protein synthesis and transport [67] and mitochondrial dysfunction [68]. Aberrant TDP-43 accumulation within the cytoplasm results in the formation of stress granules and ribonucleoprotein complexes and reduces protein synthesis within the axon and synapse [46, 69]. These translation deficits influence synaptic function and reduce the integrity of the neuromuscular junction, resulting in muscular atrophy. In human induced pluripotent stem cell (iPSC)-derived motor neurons, clearance of the axonal accumulation of TDP-43 restored the function of the neuromuscular junctions [70].

As mentioned above, a gain-of-function toxicity from cytoplasmic TDP-43 can also influence mitochondrial function. Using transgenic mice expressing wild-type hTDP-43 under a mouse prion promoter, Xu et al. [71] identified aberrant mitochondrial aggregation and dysfunctional mitochondrial mechanics from TDP-43 overexpression in the cytoplasm. Similarly, TDP-43 was found to be aggregated within mitochondria isolated from the spinal cord and cortex neurons in ALS patients [72]. In HEK-293 cells overexpressing wild-type or mutant TDP-43, TDP-43 localised within the mitochondria and disrupted mitochondrial function determined through increased mitochondrial fragmentation and reduced ATP levels [72]. Blocking TDP-43 localisation via genetic ablation of the mitochondrial localisation sequence reduced TDP-43 localisation to the mitochondria and reduced the neuronal loss and mitochondrial fragmentation compared to mutant TDP-43.

In conclusion, the combined effects of both loss of nuclear TDP-43 and the gain of toxic cytoplasmic TDP-43 aggregates should not be ruled out as the cause of TDP-43-related neurodegeneration. Knock-down of endogenous TDP-43 by siRNA in the murine spinal cord-x neuroblastoma hybrid cell line (NSC-34) was used to measure cell viability in the absence of cytoplasmic TDP-43 aggregates. Neuronal toxicity was indicated by both a significant reduction in cell viability and an increase of caspase-3 activity was found, suggestive that loss-of-function toxicity can occur without the need for TDP-43 aggregation. However, a similar result was found when TDP-43 inclusion bodies were intracellularly delivered via a plasmid expressing human TDP-43 into the NSC-34 cell line to mimic cytoplasmic aggregation. The relative contributions of loss-of-function and gain-of-function toxicity were calculated and determined to equally contribute to neuronal toxicity [73].

It has been proposed that another mechanism of TDP-43-associated neurodegeneration involves the expression of ERVs, which were previously thought to remain dormant within the genome. This mechanism is discussed further below.

## Neuroinflammatory Involvement in ALS

### The Role of Inflammation in ALS Neurodegeneration 

The impact of inflammation has been well established in ALS with dysregulation of inflammatory cytokines in ALS patients, involvement of astrocytes and microglia, and T lymphocyte infiltration into the CNS linked to ALS disease progression [74]. Animal models of ALS including *SOD1*, *C9orf72* and *TARDBP* also have dysregulated inflammatory processes, as seen in human ALS [14, 75, 76]. Transgenic mice with a loss-of-function *C9orf72* mutation had increased inflammatory cytokine expression within plasma and reduced survival rates compared to wild-type controls [77]. Furthermore, a TDP-43^Q331K^ mouse model was used to investigate the inflammatory processes with the transgenic TDP-43 mice showing increased microglial activation that correlated with motor deficits and subsequent increased progression of neurodegeneration compared to WT mice [78]. Modulation of the inflammatory processes evident in these animal models has provided evidence for slowing motor neuron degeneration and extending animal survival. For instance, cytotoxic CD8 T cells infiltrate the CNS selectively destroying motor neurons in mutant *SOD1*^G93A^ mice and increase the expression of interferon-γ (IFN-γ) [79]. Removal of this cell population via genetic ablation results in a slowing of this selective motor neuron degeneration. While the role of the immune system has been explored in the more common forms on inherited ALS, less frequent mutations in *OPTN*, *SQSTM1*, *VCP* and *TBK1* are also associated with inflammation [14, 80]. In addition, patients with sporadic ALS exhibit an activated immune phenotype including changes in cytokine concentrations including TNF-α, IL-1β, IL-4, IL-6 and IL-10 in serum [81–83]. Other inflammatory markers that can be detected within CSF, serum or urine of ALS patients includes monocyte chemoattractant protein 1 (MCP-1), C-reactive protein (CRP) and neopterin [83–85].

### Dysregulation of the cGAS/STING Pathway Influences Immune-Mediated Neurodegeneration in ALS

The cyclic guanosine monophosphate-adenosine monophosphate (cGAMP) synthase (cGAS) and stimulator of interferon genes (STING) pathway (cGAS/STING) pathway has been implicated in neuroinflammation-mediated neurodegeneration [86, 87]. cGAS detects danger signals such as double-stranded DNA within the cytoplasm and triggers the formation of cyclic cGAMP. cGAMP binds to STING and subsequently activates TBK1 resulting in phosphorylation of interferon regulatory factor (IRF) 3, IRF7 and release of nuclear factor kappa-light-chain-enhancer of activated B cells (NF-κB) from the cytoplasm. These transcription factors move to the nucleus and subsequently induce transcription of mRNA for multiple inflammatory factors such as IL-6 and TNF-α and interferons (IFNs) including IFN-α and IFN-β and released from the cell [88, 89]. Translation and release of IFN-α and IFN-β from these cells can act on neighbouring cells via the IFN-α/β receptor (IFNAR) to activate the Janus-associated kinase (JAK) and signal transducer and activator of transcription (STAT) pathway and induce transcription for interferon-stimulated genes (ISGs) [90]. Under normal physiological conditions, the cGAS/STING is neuroprotective and produces an immune response to clear unwanted pathogens and prevent cell death [87]. However, aberrant activation of this pathway has been linked to neurodegeneration, where increased IFN production results in faster disease progression [91, 92]. ALS mouse models have been used to investigate the role of cGAS/STING in neurodegeneration. In *C9orf72*^−/−^ mice, there is an upregulation of type 1 IFNs resulting in systemic CNS inflammation due to increased cGAS-STING pathway signalling [93]. Through STING^−/−^ in a neurodegenerative disease model, Nazmi et al. [94] proposed a STING-dependent toxic increase in IFNs, resulting in neurodegeneration through microglial phenotype modulation. Furthermore, a recent investigation has demonstrated that TDP-43 cytoplasmic mislocalisation results in mitochondrial DNA release that also activates the cGAS/STING pathway, resulting in the upregulation of NF-kB and IFN pathways [86]. In contrast, inhibition of STING using a validated STING inhibitor, H-151 [95] in ALS patient derived iPSCs and a TDP-43 mouse model normalises IFN levels, resulting in reduced neuronal loss and improved motor performance in mice [86], providing further evidence for the role of inflammation in the propagation of neurodegeneration in ALS (Fig. 2).

To further outline the role of the immune response in neurodegeneration, TBK1 mutations have also been linked to ALS in a small number of familial ALS cases [96, 97]. TBK1 is involved in the cGAS/STING pathway and induces IFNs while also being involved in autophagy mechanisms [98]. The dysregulation of TBK1 could be contributing to neurodegeneration through disrupted autophagy resulting in aberrant protein aggregation or through increased neuroinflammation from activation of the inflammatory pathways involving TBK1 [99].

### Non-neuronal Cells and Release of Pro-inflammatory Cytokines in ALS

Neuroinflammation in ALS includes the activation of microglia and the polarisation of microglia into two different phenotypes, either pro-inflammatory, M1, or anti-inflammatory, M2 [14]. In early stages of ALS, activated microglia produce a neuroprotective response with production of anti-inflammatory cytokines, such as IL-4 and IL-10, and are referred to as M2 microglia [100, 101]. Further into ALS disease progression, microglia become activated into an M1 phenotype with neurotoxic properties, releasing pro-inflammatory cytokines including IL-1β, TNF-α, IL-6 and IL-18 [102, 103]. As the disease progresses, levels of pro-inflammatory cytokines including TNF-α and IL-6 are increased in the blood and CSF from ALS patients compared to healthy controls or patients with other neurological diseases such as Parkinson’s disease [104–106]. TNF-α also mediates the activation of NF-κB which has apoptotic and neurotoxic properties, with increased activation of the NF-κB signalling pathway in ALS, driving further inflammatory cytokine release [74, 107]. TDP-43 and SOD1 aggregates within microglia are likely to induce a pro-inflammatory M1 phenotype due to increased NF-κB signalling pathways and NLRP3 inflammasome [108, 109].

Astrocyte-mediated neurotoxicity has been proposed to be caused by protein aggregation such as mutant SOD1 and TDP-43 [110, 111]. Furthermore, astrocytes may contribute to neurodegeneration through alteration of secreted factors [112]. In healthy function, astrocytes provide the surrounding motor neurons with neurotrophic factors such as brain-derived neurotrophic factor (BDNF) [113]. In ALS, astrocytes release toxic factors such as nitric oxide, transforming growth factor β1 (TGF-β1) and pro-inflammatory cytokines to the surrounding motor neurons and microglia [114, 115]. Overexpression of astrocyte-derived TGF-β1 in SOD1^G93A^ mice was shown to reduce the neuroprotective state of microglia and resulted in faster disease progression [116]. Moreover, an astrocyte cell line treated with CSF from ALS patients showed impaired regulation of nitric oxide and release of pro-inflammatory cytokines, IL-6 and TNF-α compared to control CSF and reduced release of neurotrophic factors [117]. In the pro-inflammatory state, T-helper type 1 cells also release IFN-γ which can further activate IRF-1 and NF-κB [118, 119].

In conclusion, while the exact mechanism of neuroinflammation-mediated neurodegeneration remains unknown, it is proposed to occur through a perpetual cycle of motor neuron death and sustained microglia and astrocyte activation with neurotoxic pro-inflammatory cytokine increases (Fig. 2). Cell-to-cell spread of toxicity occurs between non-neuronal cells and surrounding motor neurons to propagate neurodegeneration [120, 121]. The use of anti-inflammatories has been used to target neuroinflammation-mediated neurodegeneration in vitro including tocilizumab, an IL-6 receptor antagonist [122], and lenalidomide and thalidomide, a TNF-α antagonist [39, 123]. However, a phase II clinical trial of thalidomide in ALS patients did not show any differences in disease progression according to the ALS functional Rating Scale Revised (ALSFRS-R), compared to historical controls, and no significant changes in serum levels of TNF-α were determined [124]. A phase II clinical trial of an immune regulator, NP001, identified slower progression of ALS in patients with higher C-reactive protein levels at baseline but failed to reach significance in the whole cohort [125]. Targeting other players involved in neuroinflammation, such as the cGAS/STING pathway (Fig. 2), has been proposed as another potential therapeutic avenue for ALS, with STING inhibitors already in development [126, 127].

## Endogenous Retroviruses are Associated with the Progression of ALS

### Retrovirus Structure

Retroviruses are enveloped, positive-sense single stranded RNA viruses [128]. Retroviruses use an RNA-dependent DNA polymerase (RdDpol), termed reverse transcriptase (RT) that enables transcription of their viral RNA to viral DNA during replication. This is a unique property of some viruses and not a normal function found in eukaryotic cells. Instead, in eukaryotic cells, transcription of cellular genes converts DNA to RNA by DNA-dependent RNA polymerase (DdRpol) [129]. Retroviruses that are transmitted between individuals are considered as exogenous retroviruses. Two pathogenic exogenous retroviruses that infect humans are HIV and human T cell leukaemia virus type 1 (HTLV-1) [130]. The retrovirus particle consists of an RNA genome packaged with replication machinery, including integrase and RT inside the capsid core and surrounded by the envelope containing viral glycoproteins and lipid derived from cell membranes. When an exogenous retrovirus infects a cell, the genomic RNA is reverse transcribed into double-stranded DNA in the cytoplasm, that then moves to the nucleus and integrates into the chromosome of the host cell forming a provirus.

The proviral DNA genome consists of *gag*, *pol* and *env* coding regions, flanked by long terminal repeats (LTRs)*. Gag* (group-specific antigen) encodes structural proteins including the capsid, matrix and nucleocapsid; *pol* encodes the enzymatic functions of the virus, viral protease, RT and integrase; and the *env* encodes the surface and transmembrane glycoproteins, gp120 and gp140 [129]. Complex retroviruses also contain accessory genes such as *tat* within the HIV genome, encoding a transcriptional activator*.* Each LTR consists of a unique 3′ region (U3), a repeat (R) and unique 5′ region (U5). The U3 region of the LTRs serves as the viral promoter regions controlling gene expression. The R region contains the trans-activation response element (TAR), which interacts with viral tat protein during transcription and recruits cellular factors to enhance viral gene transcription [129].

### Endogenous Retroviruses: Structure and Function

**Fig. 1:**
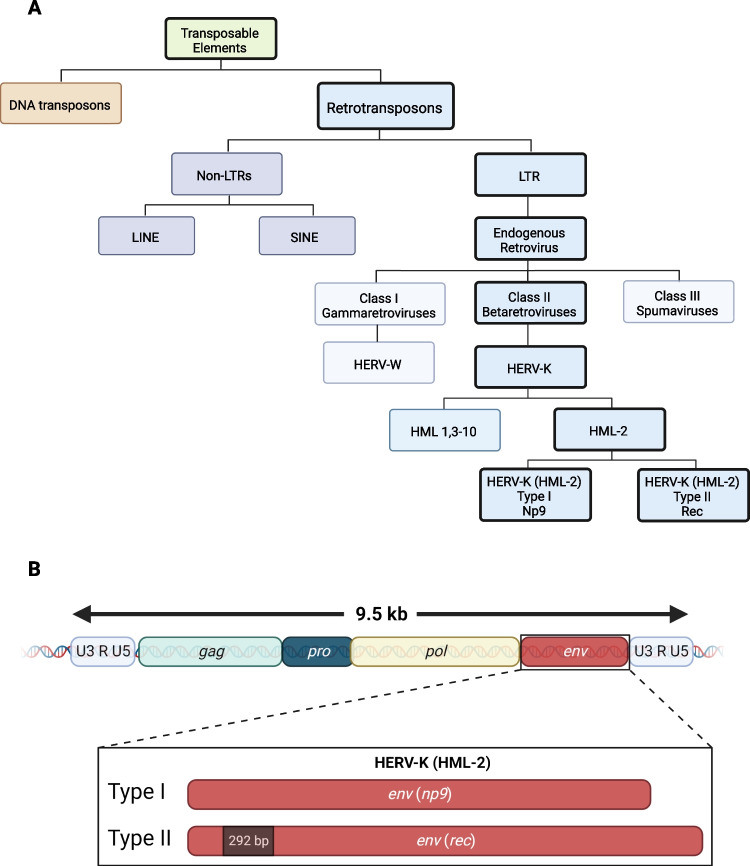
Human endogenous retrovirus type K (HERV-K) elements in the human genome. **A** HERV-K is a Class II betaretrovirus that is long terminal repeat (LTR) containing retrotransposons present within the human genome. HERV-K is distinct from other endogenous elements such as DNA transposons, the non-LTR retrotransposons such as long interspersed nuclear elements (LINE) and short interspersed nuclear elements (SINE). HERV-K is related to other LTR-containing endogenous retroviruses such as HERV-W (a Class I gammaretrovirus) and the Class III spumaviruses. The HERV-K family is further subdivided into 10 human mammary tumour-like (HML) elements, where HERV-K (HML-2) is further grouped into type I (np9) and type II (rec) based on the envelope sequence, as shown in (**B**) HERV-K (HML-2) represents full-length provirus, approximately 9.5 kb, with a capacity to generate infectious virus by virtue of the presence of 2 complete LTR’s containing U3, R and U5 regions that flank the viral structural proteins gag, pol and env. type I env region encodes the accessory protein np9, while type II harbours an additional 292 base pair region in the env ORF and encodes the accessory protein Rec. Adapted from Li et al. [134]. Figure made in BioRender

ERVs are a type of transposable element which is a type of mobile genetic element that can move to other locations in the genome. Transposable elements are classified as DNA transposons or RNA transposons. Based on the presence of LTRs, retrotransposons are further classified into non-LTRs including short interspersed nuclear elements (SINE) and long interspersed nuclear elements (LINE) or ERVs with LTRs. LTR retrotransposons can be transcribed from the host cell genome into ERV RNA, then, in the same cell, reverse transcribed back into double-stranded DNA and re-integrated into another site of the host genome [131, 132]. This would have the potential to be damaging to the host cell genome, and hence many species, including humans, have cellular processes to restrict this from happening [133]. ERVs are then classified into three different classes based on their homology to exogenous retroviruses genera. Class I encompasses gammaretroviruses, class II encompasses betaretroviruses and Class III spumaviruses [131]. The relationship between classification of transposable elements and ERVs is seen in Fig. 1A. One type of ERV, HERV-K (HML-2) and its association with ALS is discussed further below.

While exogenous retroviruses are capable of horizontal transmission from person to person and produce infectious virions, ERVs, in contrast, are traditionally not thought to produce infectious virions and are not horizontally transmitted. Instead, ERV’s have integrated into the host genome and are vertically transmitted through the germ lines [131, 135]. The process of germ-line integration of ERVs occurred millions of years ago with the subsequent process of endogenization. After endogenization within the genome, the virus no longer produces an infectious particle and lacks the capacity to infect as an exogenous retrovirus [136]. Eventually, ERVs will be fixed within the genome and inherited within every member of the species. The koala retrovirus is believed to be an example of a current exogenous retrovirus in the process of endogenization in the koala population [137]. In humans, ERVs compose 8–10% of the human genome, usually thought to be transcriptionally silent and lack the ability to transpose [131, 138]. In contrast, 10% of the mouse genome is comprised of endogenous retroviruses and, unlike human ERVs, most murine ERVs remaining transcriptionally active [139, 140].

### HERV Benefit and Role in Disease

Most human endogenous retroviruses (HERVs) are not transcribed and contain deletions and mutations resulting in a lack of functional protein production and lack the components required for a functional virus [141]. Recently, however, transcriptional activation of ERV elements in humans has been proposed as a causative factor or progressive factor for a multitude of diseases, including ALS [134, 142–144]. One type of HERV, HERV-W, does have a physiological benefit to the host with an important placental protein, syncitin-1, encoded by the HERV-W envelope gene [145]. Syncitin-1 aids in trophoblast fusion and is a necessary step in the healthy formation of the placenta [146]. Abnormal expression of syncitin-1 has been associated with pregnancy-related disorders, such as pre-eclampsia and other placenta-related pathologies [147]. While this type of HERV-W plays an important role in placental development, HERV-W expression has also been associated with multiple sclerosis (MS) pathology [148]. Several studies have identified increased levels of HERV-W env protein within brain tissue and PBMCs from MS patients compared to healthy controls [149–152]. The reactivation of HERV-W and the association with MS have been proposed to be caused by an exogenous viral infection from Epstein-Barr virus [148, 153, 154].

Thus, while some HERVs have physiological importance, dysfunctional expression of these HERVs can have detrimental effects on the host. Other HERVs have been correlated with a variety of diseases: HERV-K, HERV-E, and HERV-W are associated with cancers such as ovarian cancer and breast cancer [155–158], HERV-W and HERV-K are associated with autoimmune diseases [155, 159], and HERV-W is associated with schizophrenia [160, 161].

The evolutionarily youngest ERV to enter the human genome is HERV type K (HERV-K), which is predicted to have endogenized into the human genome approximately 700,000 years ago [162]. HERV-K is a class II ERV and is referred to as type K due to the use of lysine (single amino acid code, K) tRNA as a reverse transcription primer. HERV-K is further classified into 10 families denoted from HML-1 to HML-10 based on their similarity with the mouse mammary tumour virus (MMTV), a prototype used for comparison when new HERVs first became to be described [163]. Human endogenous mouse mammary tumour virus like-2 (HML-2) is the best preserved HERV-K element, maintaining the capability of encoding viral proteins such as the env protein [164]. The delineation of HERV-K and HML subtypes is shown in Fig. 1A, and the HERV-K proviral genome structure can be seen in Fig. 1B [155, 165].

Two LTR regions are also present on a portion of other HERV families [165, 166]. HERV-K (HML-2) is further classified into two types based on the expression of accessory genes. HML-2 type I proviruses have a 292 bp deletion within *env* and encode accessory protein, Np*9* while type II proviruses encode accessory protein, Rec [167]*. Rec* is similar to HIV-1 *Rev* accessory gene, a protein that is involved in RNA splicing. While the biological role of these proteins is still unclear, mRNA transcripts for Rec and Np9 from multiple HERV-K loci have been found in many human tissue types [168].

Many of the HERVs that exist in the human genome are in the form of solitary LTRs [19, 20]. However, some HERVs, as described above for HERV-K and HERV-W, retain intact open reading frames (ORFs), with the ability to produce functional proteins [169, 170]. Approximately 950 solitary LTRs have been described in the human genome [163] with 17 identified full-length HERV-K [171–173]. Both the solo LTRs and the HERV-K proviral elements capable of producing RNA and proteins have been implicated in diseases [173].

### Human Endogenous Retrovirus Type K is Associated with ALS

The link between ALS and retrovirus activity was first identified in 1975, through discovery of RT activity in brain tissue of two ALS patients [174]. Further studies confirmed this finding with elevated RT levels in serum and CSF of ALS patients without exogenous retroviral infection [175–177]. Andrews et al. [175] demonstrated increased RT levels in 59% of the 56 ALS patients compared to 5% of the 58 controls. In a separate cohort of 14 ALS patients, RT activity in serum was detected in 47% of the ALS patients compared to 18% in the controls. However, RT activity was also elevated in blood relatives of the ALS patients in this cohort [18, 176]. Rare cases of an ALS-like syndrome were observed in patients infected with exogenous retroviruses such as HIV-1 [178, 179]. Additionally, these motor symptoms were observed to be reversed in the HIV positive patients after they were inititated on antiretrovial therapy (ART). This association between HIV and development of ALS-like motor symptoms was proposed to occur through activation of a specific endogenous retrovirus, HERV-K with a reduction in the levels of HERV-K DNA within the plasma after ART [19, 180].

Experimental studies further supported the proposed association between ALS and ERVs. Hadlock et al. [181] evaluated the immunoreactivity of ALS patient serum to HML-2 gag protein. Their study observed that ALS patient serum had greater than fivefold higher IgG reactivity to recombinant gag (57% vs 11% in ALS patients and age-matched controls respectively). This finding suggests that HML-2 gaga can induce an antibody response in ALS patients and the involvement of ERVs in Immune-mediated ALS has been proposed [178].

Recently, an antibody response to specific epitopes of HERV-K (HML-2) env has also been demonstrated with a greater antibody response in ALS patients compared to age- and sex-matched controls [182].

Furthermore, HERV-K pol transcripts, measured through quantitative real-time PCR, in the brain tissue from the prefrontal cortex, sensory cortex and occipital cortex of 28 ALS patients were compared to levels in brain tissue from people who succumbed to other diseases. These HERV-K pol transcripts were found to be significantly higher in ALS patients than age-matched controls [21]. Post-mortem cortical brain tissue analysis from 11 ALS patients using RT-PCR identified increased expression of 3 HERV-K genes, *gag*, *pol* and *env* compared to control brain tissue [134]. Transfection of a construct to express the HERV-K *env* gene in human neuronal cultures, derived from iPSCs, has demonstrated the toxicity env, with reduced viable neuronal cell number after *env* transfection [134]. Similarly, Steiner et al. [22] found an increase of HERV-K env protein in CSF from 11 out of 15 ALS patients measured through immunocapillary Western blot and in only one healthy age-matched control. The authors also demonstrated the neurotoxic properties of HERV-K env protein through intracerebral injection of recombinant env protein into mice, showing a reduction in neuronal cell number 1-week post-injection compared to control injection. These results provide further support for a role of HERV-K in neurodegeneration in ALS.

### Association Between HERV-K, TDP-43 and Inflammation May Cause neurodegeneration in ALS

The association between HERV-K and ALS pathology is proposed to occur through an interaction with TDP-43. Importantly, chromatin immunoprecipitation identified 5 binding sites for TDP-43 on the consensus sequence of HERV-K LTR [134]. This suggests that TDP- 43 may be involved in HERV-K transcriptional regulation, discussed previously in the “TDP-43 structure and function” section [47]. HERV-K RT expression is positively correlated with TDP-43 protein levels within the cortical brain tissue and human neuronal cells from iPSCs supporting this regulatory link [21, 134]. An in vitro study with cultured human neural progenitor cells transfected with a construct to express mutant human TDP-43 identified an increase in HERV-K RT mRNA levels in the transfected cells compared to untransfected cell [183].

HERV-K has also been shown to influence TDP-43 expression and aggregation [134, 184]. Ibba et al. [185] proposed an association between HERV-K and TDP-43 when disruption of HERV-K *env* throughout the genome resulted in a decrease in TDP-43 mRNA and protein levels in human prostate adenocarcinoma cells. While previous findings have identified TDP-43-dependent increases in HERV-K expression [183], the above finding demonstrates the inverse relationship in that HERV-K is capable of regulating TDP-43 mRNA and protein expression levels, suggesting a positive feedback loop of TDP-43 and HERV-K activation [185, 186]. Chang and Dubnau [187] established a drosophila model expressing TDP-43 within glial cells to elucidate the mechanisms of ERV-TDP-43 involvement in neuronal damage. In this model, glial TDP-43 protein aggregates increased the expression of drosophila ERVs within the glial cells. This increased ERV expression within glia resulted in increased cellular release of neuronal toxic factors that induced DNA damage and neuronal death in surrounding neurons. These studies provide evidence for a self-perpetuating feedback loop between HERV-K and TDP-43 as a potential mechanism of neurodegeneration in ALS.

As outlined above, there is increased activity of transcription factors that drive inflammatory mediator production including IRF-1, IRF-3 and NF-κB in ALS [74, 188]. The TDP-43 promoter has binding sites for IRF-1, IRF-3 and NF-κB, suggesting the role of activation of these transcription factors in driving increased expression of TDP-43 and potentially TDP-43 proteinopathy. Similarly, HERV-K expression is induced by inflammatory mediators within neurons and non-neuronal cells. HERV-K LTR consensus sequences contain two interferon-stimulated response elements, which will activate HERV-K expression when activated by type I IFN signalling and activation of the JAK/STAT pathway [189]. Furthermore, IFN-γ has been experimentally shown to increase transcription of HERV-K *gag* and *pol* determined by q-RT-PCR and increased RT activity in an astrocytic cell line [190]. Additional evidence for the association between ERVs and inflammation has been demonstrated in vivo [23, 191, 192]. Genetic deletion of known ERV repressor, Trim28, in mice during development resulted in increased ERV expression in the adult cortex of the mice and increased microglia activation, suggestive of a pro-inflammatory environment in the brain [23].

In support of the relationship between inflammation and ERVs, NF-κB is also thought to induce HERV-K expression. Manghera et al. [24] demonstrated increases in HERV-K expression, measured by levels of HERV-K RT activity, when transfected with constructs expressing NF-κB in human neural progenitor cells. Neuroinflammatory mediators such as TNF-α and NF-κB also increase TDP-43 expression which can drive further HERV-K expression and further neuroinflammation. This pathway may result in the neurodegeneration observed in ALS, with cell-to-cell spread of toxicity [193, 194].

### Treatment of HERV-K Associated ALS Through Antiretroviral Therapy

As described, HERV-K has been implicated in the causation and perpetuation of the signals that drive neurodegeneration in ALS. This and the early clinical anecdotal findings of improved ALS-like symptoms in HIV patients on ART led to the proposal that targeting ERVs could be used as a treatment for ALS, through ART designed to target HIV [180, 195]. Two early clinical trials investigated the effect of two different antiretrovirals in ALS patients, a nucleoside reverse transcriptase inhibitor (NRTI), Zidovudine and a protease inhibitor, Indinavir [196, 197]. Neither study identified any slowing of disease progression, although low sample sizes and poor adherence due to the advancing ALS symptoms resulted in inconclusive results. An in vitro study demonstrated the ability of an NRTI, abacavir, to inhibit HERV-K using a pseudotyped HERV-K with infectious capabilities. Pseudotyped HERV-K-infected HeLa cells were treated with abacavir, and HERV-K RT levels were examined through RT assay and determined to be significantly reduced [195]. Interestingly, abacavir was more potent against HERV-K than HIV as determined by significantly lower IC_50_ and IC_90_ concentrations of the drug. Triumeq is an example of combination ART that is widely used for treatment of HIV which consists of two NRTIs, abacavir and lamivudine and an integrase inhibitor, dolutegravir, all of which are capable of penetrating the CNS [198–200].

**Fig. 2:**
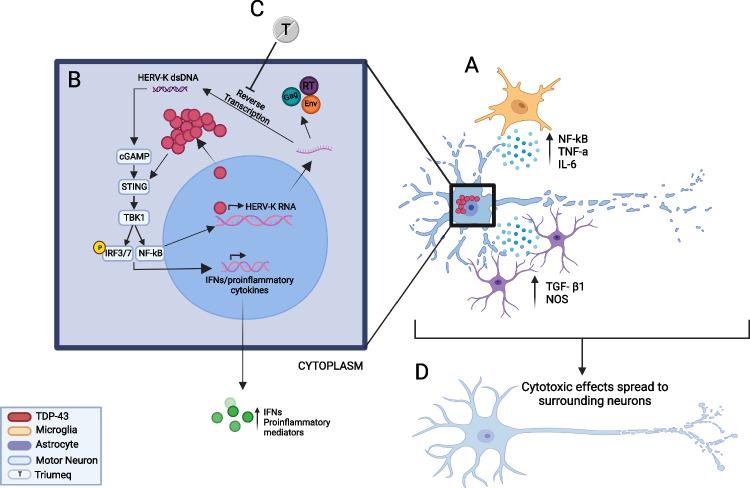
Depiction of the proposed interactions of HERV-K, TDP-43 and inflammatory mediators in the process of neurodegeneration in ALS. A Astrocytes and Microglia release pro-inflammatory cytokines that can induce TDP-43 cytoplasmic mislocalisation in neurons. **B** Mislocalisation of TDP-43 to the cytoplasm de-represses HERV-K transcription which leads to the production of HERV-K RNA. The HERV-K mRNA is translated into HERV-K proteins including Gag, envelope (env) and the reverse transcriptase enzyme (RT) from the pol gene. The RT enzyme acts to reverse transcribe the HERV-K into double-stranded DNA (dsDNA). Cytoplasmic dsDNA is a danger signal that is recognised by and activates the cGAS/STING pathway that subsequently activates TBK1 resulting in phosphorylation of IRF3, IRF7 and release of NF-*κ*B from the cytoplasm. These transcription factors move to the nucleus and subsequently induce transcription of mRNA for multiple inflammatory factors and interferons. Additionally, NF-*κ*B can further drive HERV-K transcription. **C** Triumeq contains two RT inhibitors which could act on inhibiting the reverse transcription of HERV-K RNA into dsDNA to prevent the activation of cGAS/STING pathway. **D** This would be predicted to reduce the release of inflammatory mediators and prevent the spread of toxicity between neurons. IRF3/7, interferon regulatory factors 3 and 7; NF-κB, nuclear factor kappa-light-chain-enhancer of activated B cells; TNF-α, tumour necrosis factor alpha; TGF-β1, transforming growth factor β1; NO, nitric oxide; P, phosphorylation; TBK1, tank binding kinase 1; IL-6, interleukin 6; T, Triumeq. Image made in BioRender

Theoretically, the two reverse transcriptase inhibitors within Triumeq, abacavir and lamivudine could inhibit the formation of HERV-K double-stranded DNA inside cells where HERV-K has been activated. cGAS/STING is a cell sensor that detects dsDNA in the cytoplasm as a danger signal, and an activation of the cGAS/STING pathway has been suggested to occur in ALS [86]. Thus, NRTI actions to inhibit HERV-K RT activity and reduced production of dsDNA in the cytoplasm that would subsequently activate cGAS/STING can be envisioned as a mechanism that may underpin the therapeutic success of ART and agents such as Triumeq. It would be expected that such a treatment would reduce production of inflammatory mediators and to slow progression of ALS (Fig. 2).

Recently, the de-repression of HERV-K was proposed to be involved in ageing, with increased HERV-K gag, pol and env transcript levels and protein levels in senescent human mesenchymal progenitor cells (hMPCs) compared to phenotypically young cells [201]. The increased HERV-K levels in these cells coincided with increased activation of cGAS/STING. Senescent hMPCs treated with abacavir showed reduced HERV-K DNA and reduced levels of inflammatory cytokines IFN-α, IFN-β and IL-1β measured through q-PCR compared to vehicle treated senescent hMPCs. Antiretrovirals have also been shown to have anti-inflammatory properties, decreasing immune activation and inflammatory mechanisms as determined by reductions in TNF-α, IL-6 and IFN-γ in patients with HIV [202, 203].

A phase IIa clinical trial for Triumeq as a treatment for ALS has recently been completed [204]. This clinical trial involved investigating the safety and tolerability of Triumeq in 40 patients with ALS across 24 weeks of treatment. During the 24 weeks, the amyotrophic lateral sclerosis functional rating scale—revised (ALSFRS-R) was used as a primary outcome measure of disease progression along with secondary measures of respiratory function, grip strength and the biomarkers, p75^ECD^, neurofilament-light and phosphorylated neurofilament heavy. Levels of serum HERV-K were also measured through droplet digital PCR.

The results of the study showed patients on Triumeq treatment had a slower clinical decline as measured by the ALSFRS-R compared to pre-treatment. HERV-K DNA serum levels were significantly decreased over the treatment course [204, 205]. This research has progressed to a phase III clinical trial to further assess the efficacy of Triumeq in halting the progression of ALS and increasing survival. This will be completed with approximately 400 ALS patients from Europe, UK and Australia. While the phase IIa clinical trial has shown promise, the mechanism of action of Triumeq for use as an ALS therapeutic is still unclear. Interestingly, another antiretroviral, raltegravir, has been trialled in relapsing remitting multiple sclerosis, but unfortunately this did not produce any clinical improvement [206].

Previous studies in mice and drosophila have identified the regulation of ERVs from TDP-43 expression suggesting a similar mechanism of TDP-43 binding to ERVs as is seen with HERVs and TDP-43 [207, 208]. Furthermore, a recent study has shown the effectiveness of using antiretroviral therapy on inhibiting mouse ERVs and reducing inflammation as shown by a reduction in IL-1β and Il-6 in abacavir-treated mice compared to vehicle controls [201]. Therefore, mouse models may be useful for understanding the complex interplay between ERVs, TDP-43 and inflammation in human ALS and elucidating the benefits of Triumeq on this interaction.

## Conclusions and Future Perspectives

The causation and progression of ALS are elusive, and the current approved therapeutics for ALS have limited effect [209]. The involvement of TDP-43 and the involvement of inflammatory processes are well-established in neurodegeneration in ALS, yet therapeutics targeting these mechanisms have not shown clinical efficacy [210]. The development and discovery of therapeutics for ALS require further investigation into the pathogenesis of ALS to determine candidate targets. The involvement of HERV-K has been experimentally established to be involved in neurodegeneration and proposed to be associated with TDP-43 and neuroinflammatory mechanisms including the cGAS/STING pathway [86]. This involvement of HERV-K in ALS led to the ongoing clinical trial of antiretroviral therapy for ALS patients (NCT05193994) with earlier trials having promising effects [204]. However, the exact mechanism of the involvement of HERV-K in neurodegeneration in ALS and the relationship with TDP-43 and neuroinflammation are still unclear. Future research will need to investigate the effects of antiretroviral therapy on HERV-K, TDP-43 proteinopathy and inflammatory processes including inflammatory cytokine expression. Thus, further elucidating the functional relationship between HERV-K, neuroinflammation and TDP-43 will allow for a greater understanding of potential therapeutics to target the intersection of these mechanisms and hopefully slow or halt ALS disease progression.

## Data Availability

Not applicable
